# The languages of health in general practice electronic patient records: a Zipf’s law analysis

**DOI:** 10.1186/2041-1480-5-2

**Published:** 2014-01-10

**Authors:** Leila R Kalankesh, John P New, Patricia G Baker, Andy Brass

**Affiliations:** 1 School Of Computer Science, University of Manchester, Oxford Road, Manchester M13 9PL, UK; 2 Tabriz University of Medical Sciences, Tabriz, Iran; 3 Salford Royal NHS Foundation Trust, Stott Lane, Salford M6 8HD, UK; 4 School of Medicine, University of Manchester, Oxford Road, Manchester M13 9PL, UK; 5 Northwest Institute for Bio-Health Informatics, University of Manchester, Oxford Road, Manchester M13 9PL, UK

## Abstract

**Background:**

Natural human languages show a power law behaviour in which word frequency (in any large enough corpus) is inversely proportional to word rank - Zipf’s law. We have therefore asked whether similar power law behaviours could be seen in data from electronic patient records.

**Results:**

In order to examine this question, anonymised data were obtained from all general practices in Salford covering a seven year period and captured in the form of Read codes. It was found that data for patient diagnoses and procedures followed Zipf’s law. However, the medication data behaved very differently, looking much more like a referential index. We also observed differences in the statistical behaviour of the language used to describe patient diagnosis as a function of an anonymised GP practice identifier.

**Conclusions:**

This works demonstrate that data from electronic patient records does follow Zipf’s law. We also found significant differences in Zipf’s law behaviour in data from different GP practices. This suggests that computational linguistic techniques could become a useful additional tool to help understand and monitor the data quality of health records.

## Background

A recent survey has shown that 90% of patient contact with the National Health Service (NHS) in the UK is through General Practices and General Practitioners (GPs) [1]. Over 98% of the UK population is registered with a general practitioner and almost all GPs use computerised patient record systems, providing a unique and valuable resource of data [2]. About 259 million GP consultations are undertaken every year in the UK. However, capturing structured clinical data is not straightforward [3]. Clinical terminologies are required by electronic patient record systems to capture, process, use, transfer and share data in a standard form [4] by providing a mechanism to encode patient data in a structured and common language [5]. This standard language helps improve sharing and communication of information throughout the health system and beyond [6,7]. Codes assigned to patient encounters with the health system can be used for many purposes such as automated medical decision support, disease surveillance, payment and reimbursement of services rendered to the patients [8]. In this work we are focusing our attention specifically on the coding system used predominantly by UK GPs, the Read codes.

Read codes provide a comprehensive controlled vocabulary that has been structured hierarchically to provide a mechanism for recording data in computerised patient records for UK GPs [9]. They combine the characteristics of both classification and coding systems [10]. Most data required for an effective electronic patient record (demographic data, lifestyle, symptoms, history, symptoms, signs, process of care, diagnostic procedures, administrative procedures, therapeutic procedures, diagnosis data, and medication prescribed for patient) can be coded in terms of Read codes [11]. Each Read Code is represented as 5-digit alphanumeric characters and each character represents one level in hierarchical structure of Read codes’ tree [12]. These codes are organised into chapters and sections. For example Read codes beginning with 0–9 are processes of care, those beginning with A – Z (uppercase) are diagnosis, and those beginning a-z (lowercase) represent drugs (described further in the Methods section). Of some concern, however, is the quality of the data captured in this way.

At its heart, medical coding is a process of communication, with clinical terminologies bridging the gap between language, medicine and software [13]. Read codes can be thought of as a vocabulary for primary care medicine, providing words (terms) used to describe encounters between GPs and patients. The GPs (annotators) are attempting to encode information regarding the consultation; information that the wider community then needs to decode. The bag of codes associated with a consultation can therefore be thought of a sentence made up of words from Read, a sentence written by a GP to convey information to a range of different listeners.

One of the best known and universal statistical behaviours of language is Zipf’s law. This law states that for any sufficiently large corpus, word frequency is approximately inversely proportional to word rank. In fact, Zipf’s law is considered as a universal characteristic of human language [14] and as a wider property of many different complex systems [15] as well as human languages [16]. Zipf suggested that this universal regularity in languages emerges as a consequence of the competing requirements of the person or system coding the information (speaker) compared with the person or system trying to decode the information (listener). From the perspective of the speaker, it would be most straightforward for them to code the signal using high level, non-specific terms as these are easy to retrieve. It is more difficult to code the signal using very specific terms as this requires hunting through long lists and navigating deep into the terminology. The problem is very different for the listener. For them the problem is one of resolving ambiguity. If the data is coded using very specific terms then ambiguity is minimal and interpreting the message is straightforward. If only high level general terms are used, then it is much harder to discern the meaning of the message. In any communication system there is therefore a tension between the work being done by the speaker and the listener. Indeed, some controversial recent papers have attempted to show that Zipf’s law emerges automatically in systems that simultaneously attempt to minimise the combined cost of coding and decoding information [16-18].

Similar issues clearly arise in medical coding in which there needs to be a balance between the efforts required from the coder with those of the person interpreting and using the data. Reaching a proper balance between comprehensiveness and usability of clinical vocabularies is regarded as one of the challenges in the medical informatics domain [19].

The hypothesis we are therefore exploring in this paper is whether a Zipfian analysis of medical coding data can provide useful insights into the nature and quality of data. For example, we can ask where this balance lies across different aspects of the data medically-coded captured in GP records, information about diagnosis, information about the medical procedures applied and medication prescribed, and whether this balance is different across different general practices. We have therefore performed a computational linguistics analysis of a large corpus of anonymised Read code data from GPs in Salford to see whether such analyses might have value in understanding and characterising coding behaviour and data quality in electronic patient records. Salford is a city in the North West of England with an estimated population of 221,300. The health of people in Salford is generally worse than the English average, including the estimated percentage of binge drinking adults, the rate of hospital stays for alcohol-related harm, and the rate of people claiming incapacity benefit for mental illness. However, the percentage of physically active adults is similar to the English average and the rate of road injuries and deaths is lower.

## Methods

### The data set

For this study we took GP data from Salford. Data from 2003 to 2009 was collected from 52 General Practice groups from Salford. This data consisted of anonymised patient identifiers, anonymised GP practice identifiers and the set of Read codes collected. In total, the data set contains over 136 million Read codes derived from 34200 distinct codes. Ethical permission for this study was granted through North West e-Health. Table 1 shows an example of a set of Read codes and demonstrates the way in which specificity increases with code depth.

**Table 1:** An example of the 5-byte Read code that shows how the specificity of a term increases as a function of depth

**Depth**	**Read code**	**Term**
1	G	Circulatory system diseases
2	G3	Ischaemic heart disease
3	G30	Acute myocardial infarction
4	G301	Other specified anterior myocardial infarction
5	G3011	Acute anteroseptal infarction

It is straightforward to examine the datasets to determine the range of term depths that have been used in the coding process.

### Zipf’s law analysis

Mathematically, Zipf’s law can be expressed as:

$$f\;\left(r\right)=r^{-\alpha }$$

where *f*(*r*) refers to the frequency of the word with rank *r* and *a* is the Zipf’s law exponent. There are a number of different ways in which this behaviour can be represented mathematically - power law behaviour, Zipf’s law, Pareto’s law - that can be demonstrated to be equivalent [20]. For example, if P (ƒ) is the proportion of words in a text with frequency ƒ then Zipf’s law can also be expressed as:

$$P\left(ƒ\right)\sim {ƒ}^{\;–\beta }$$

It is straightforward to show that *β* and *α* are related by:

$$\beta =1+\frac{1}{\alpha }$$

Figures in this paper have been presented in the form of the Pareto distribution (named after a nineteenth century Italian economist) as they provide the most convenient form for calculating an accurate exponent. The Pareto distribution is expressed in terms of the cumulative distribution function (CDF):

$$P\left(X\geq x\right)\sim x^{-k}$$

where the distribution shape parameter, *k*, can be converted to the Zipf’s law exponent (*a*) via:

$$\alpha =\frac{1}{k}$$

and to the power law exponent (*β*) as below:

β=1+k

Pareto plots and parameter estimations were calculated using the Matlab packages plfit, plplot and, plpva developed by Clauset and Shalizi [21]. These packages attempt to fit a power law model to the empirical data and then determine the extent to which the data really can be effectively modeled using a power law. These tools provide two statistics describing the data. The first is a p-value that is used to determine the extent to which the power law model is appropriate. If the p-value is greater than 0.1 we can regard the power law to be a plausible model of our data. The second statistic produced is β, the exponent of power law.

A number of Zipfian analyses were then performed on different subsets of the Read code data within the Salford corpus. In particular we looked at the subsets of Read codes for codes to do with diagnosis, procedure and medication separately (Read codes used for diagnosis start with an upper case character (A-Z), Read codes for procedures begin with a number (0–9), and those medication with a lower case character (a-z) [22]). We were able to further subdivide the data into chapters based on the first letter of the Read code for more detailed analysis.

We also performed a number of other simple analyses to characterise the Salford corpus. We first measured the type-token ratio (TTR). The TTR is calculated by dividing the types (the total number of different Read codes) by tokens (total number of Read codes used), expressed as a percentage. In essence, this measure is equal to the number of distinct terms (Types) in the corpus divided by the total number of terms (Tokens) used [23]. A low TTR is a signal that there is a lot of repetition in the terms used, a high TTR ratio is a signal that the “vocabulary” (distinct terms) used is rich. A second analysis examined the typical depth of the terms used from the Read codes in each of the subsets of data. In a final analysis we characterised the Read code terminology itself, to how many terms at each level there were available to GPs in each chapter. We then repeated this analysis in the Salford data looking at the set of codes that were actually used from this full set. From this we were able to determine the extent to which GPs did, or did not, take advantage of the structure inherent in the terminology.

## Results

**Figure 1:**
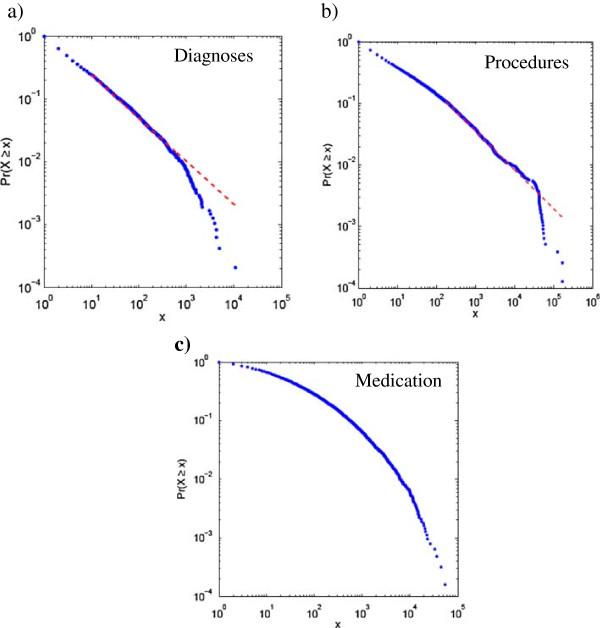
**The Pareto plots for the Salford data showing the cumulative distribution function Pr(x) plotted as a function of frequency (x) for the subset of the Read codes used in the Salford corpus. a)** diagnosis codes; **b)** procedure codes; **c)** medication codes. The data for diagnosis and procedure codes could be effectively modelled, at least in part of their range, by a power law (shown as the dotted lines in **a** and **b)**. However, there was no range on which the medication data could be modelled by a power law, **c)**.

In the first analysis, the data was split by the three Read code sections (diagnosis, procedure and medication) and the Pareto distributions and power law exponents were determined. The Pareto plots for these data are shown below in Figures 1a to c. For these data sets, the values of the power law exponent for diagnosis, procedures, and medication were 1.66, and 1.68, and 1.94, with associated Type-Token Ratios (TTRs) of 2.7%, 0.32%, 0.35% respectively. However, the data in Figure 1c was not effectively modelled by a power law (as determined by a p-value < 0.1) as there is no region of this curve that could be modelled by a straight line. A similar analysis was performed on data from specific sub trees from the diagnosis chapters. In all cases we found clear Zipfian behaviour (data not shown) for chapters in the diagnosis and procedure sections.

**Figure 2:**
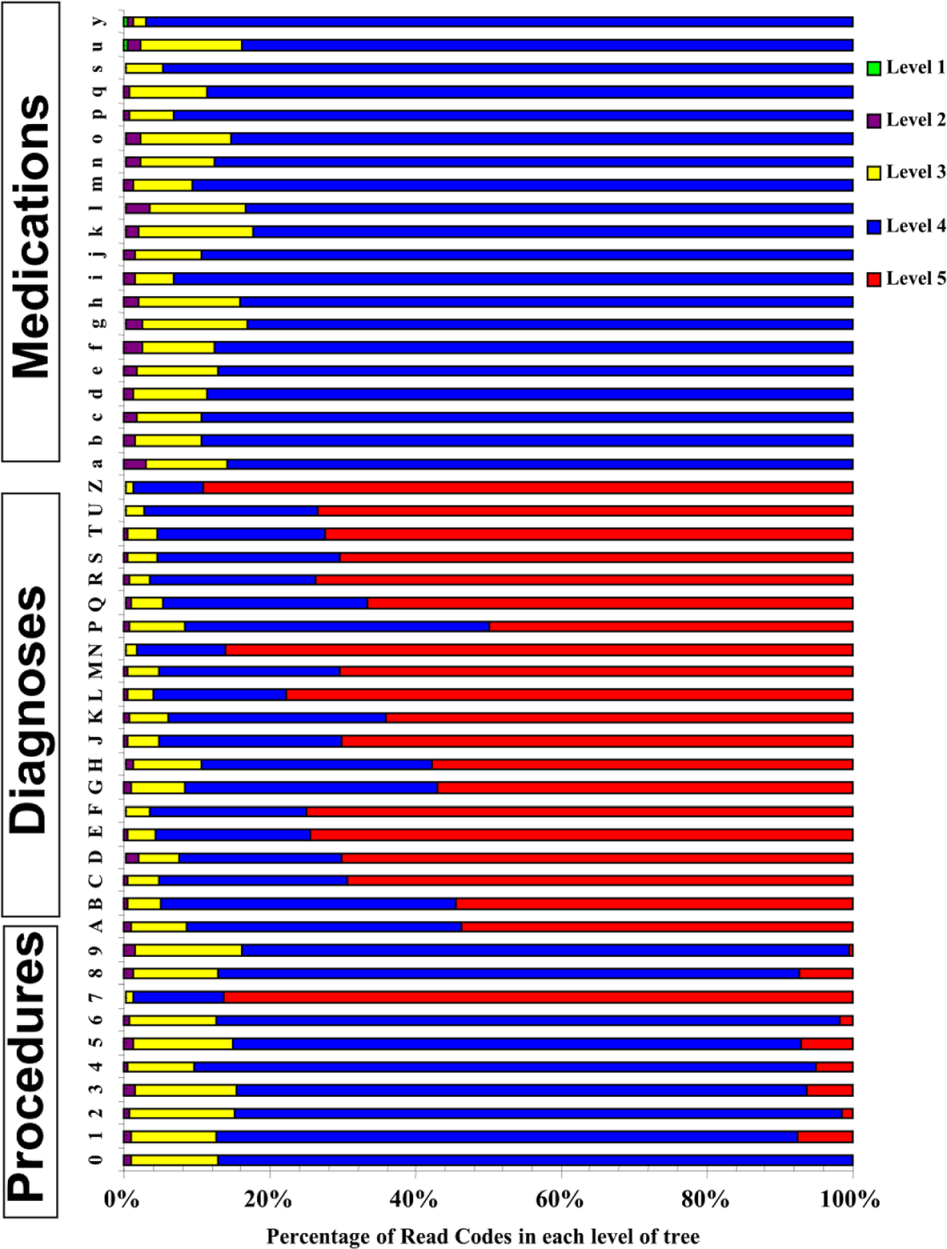
Percentage of Read codes at each level of granularity as a function of the Read code chapter.

**Figure 3:**
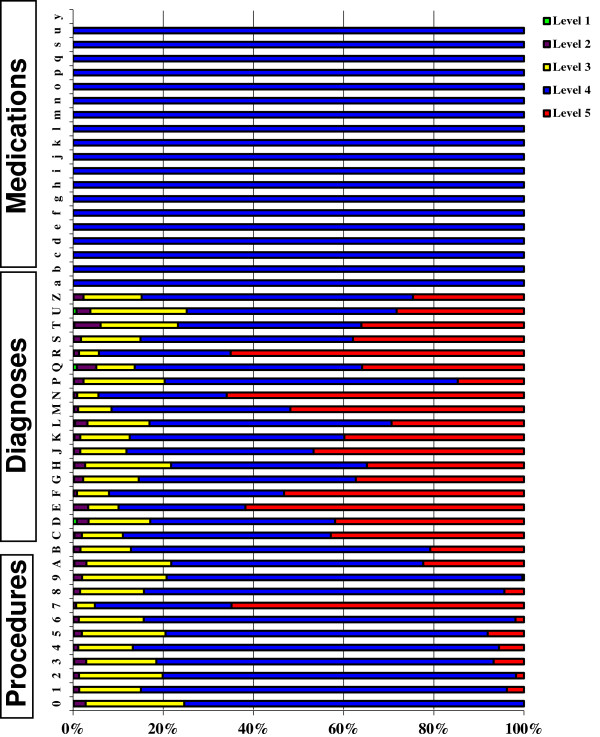
Percentage of Read codes at each level of granularity as a function of the Read code chapter as used by GPs in the Salford data set.

It is evident from Figure 1c) that the medication codes do not show Zipfian behaviour. We therefore explored the difference between the medication codes and other codes from two perspectives: the depth of the codes provided by the coding system itself for different categories of data (Figure 2), and the depth of codes used for describing different categories of data by doctors in practice (Figure 3). In some chapters of Read codes, the hierarchies are deeper than in others. For example, the highest depth of hierarchy for medication codes in the coding system is 4, whereas the highest depth of hierarchy for diagnosis and procedure codes in the coding system is 5. It is interesting to note that in the medication data all the codes used had depth 4 and that there were no codes with depths less than this. This contrasts sharply to the codes used in procedure and diagnosis which use a range of depths comparable to those provided in the Read code hierarchy. This is an indication that the medication data have been encoded in such a way that information transfer can be maximised toward satisfying decoder needs (the speaker has navigated to the roots of the hierarchy to encode the information). It can be also interpreted that the medication Read Code ‘r’ has been referred to the drug ‘d’ only if ‘r’ can be understood as referring to ‘d’ by someone other than the speaker (encoder) as a result of the communication act, an indexical reference system [24].

**Figure 4:**
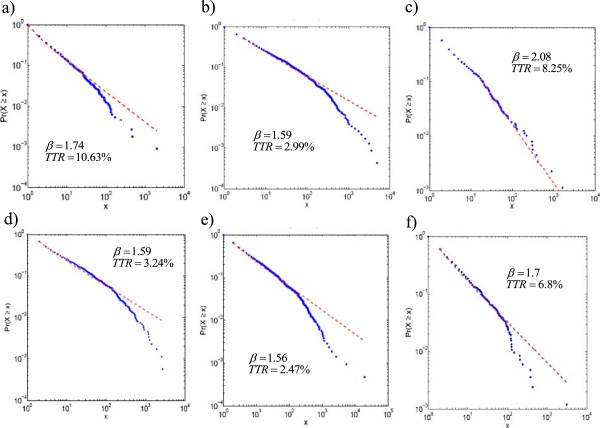
**The Pareto plots for diagnosis Read codes used from six separate GP practices from 2003–2006 (denoted as a to f).** On each figure we also show the measured values of β, the measured Zipf’s law exponent, and the TTR, the type-token ratio.

The data were then analysed as a function of the anonymised GP practice identifier. The typical values of *β* in the data ranged from 1.56 to 2.08. Percentage of type token ratio for aforementioned GP practices ranged from 2.47% to 10.63%. This strongly suggests that the range of coding vocabulary used by different GP practices varies considerably in its richness and degree of repetition. In most of the graphs, two different regions could be recognised, a linear region on the left hand side (the more uncommon terms) that fits the power law behaviour and a second region of higher frequency terms; the transition between these region being the point at which the graph deviates from the fitted line (Figure 4). A similar pattern has been observed in a Zipfian analysis of the British National Corpus (BNC) [25]. In the BNC corpus, the region of more commonly deployed codes was defined as a core vocabulary – the words commonly used - and the region of less commonly used codes as a peripheral vocabulary – words more rarely used. A similar interpretation can be made of the data from the medical records. Despite difference in the value of exponents, all plots have one feature in common: average depth of codes in the region of “core vocabulary” is smaller (range 3.3-3.7) than that found in the regions of “peripheral vocabulary” (range 3.6-4.3). The analogy with language would be that the codes near the top of the Read code hierarchy constitute a core, commonly used, vocabulary, whereas the more specialist terms found deeper in the hierarchy relate to a more peripheral and rarely used vocabulary.

## Discussion and conclusions

Within the Salford corpus, the usage of Read codes for diagnosis and process show a power law behaviour with exponents typical of those seen in natural languages. This supports the hypothesis being made in this paper that there are overlaps between the processes involved in describing medical data (terms chosen from a thesaurus to describe an encounter between a patient and a GP) and human communication (words chosen to describe a concept to a listener). This was not only true of the complete data sets; it was also seen to be true of the data from the specific chapters.

However, the story is not completely straightforward. There was one section of data captured by Read codes that showed a very different behaviour, namely the medication data. These data showed no evidence of Zipf’s law behaviour and it would appear that the principle of reaching a balance between the encoding and decoding costs has broken down. The pattern of code use from the hierarchy of Read codes is very different for the medication data compared with process or diagnosis code. All Read codes used by GPs for encoding the drug information is from the highest level provided by the hierarchy of Read Code System. This would suggest that, in the case of medication information, doctors attribute very high value to creating minimal ambiguity in the message to the maximum extent the coding system allows them. This is perhaps unsurprising as the prescription data are an input for another health care professional in the continuum of care (pharmacist) and any ambiguity in the case of this sensitive data could be harmful or fatal to a patient. The exact match between expression and meaning by someone other than encoder is critical. From this perspective, medication data seem to behave as an indexical reference in which an indexical expression “e” refers to an object “o” only if “e” can be understood as referring to “o” by someone other than the speaker as a result of the communicative act.

It is also the case that not all GPs use language in the same way. It is known that capture of diagnosis information is very variable between different GP practices [26]. At this stage, it is difficult to provide detailed explanation reasons for this. It could be that this reflects a difference in the populations being served by each GP; however we do not have the information available to us in this study to allow us to address this. However, it is suggestive that this form of computational linguistic analysis could provide useful information on the quality of data being captured from different GP surgeries. There is a significant body of work in language processing looking at power law exponents and how they change with different qualities of language, an analysis that could well have useful analogies for these data. At this stage we do not have the information to determine the extent to which the signal mirrors the quality of the data capture by the GPs, but this is clearly something that would warrant further study.

Therefore, there are aspects of GP records that behave very like a language and for which it would be appropriate to apply the methodologies of computational linguistics. Our hope is that the development of such methods could provide important new tools to help assess and improve the quality of data in the health service.

## Abbreviations

BNC: British National Corpus; CDF: Cumulative Distribution Function; GP: General Practitioners; NHS: National Health Service; TTR: Type-Token Ratios.

## Competing interest

The authors declare that they have no competing interests.

## Authors’ contributions

LRK performed the analyses and helped draft the paper. JPN and JP provided the data sets for analysis and helped in the data interpretation. AB conceived of the study, participated in its design and coordination and helped to draft the manuscript. All authors read and approved the final manuscript.
